# Whole-genome analysis revealed the growth-promoting mechanism of endophytic bacterial strain Q2H1 in potato plants

**DOI:** 10.3389/fmicb.2022.1035901

**Published:** 2022-12-01

**Authors:** Yuhu Wang, Qianqian Zhao, Zhenqi Sun, Yahui Li, Hongtao He, Yuanyu Zhang, Xiangdong Yang, Dong Wang, Baozhu Dong, Hongyou Zhou, Mingmin Zhao, Hongli Zheng

**Affiliations:** ^1^College of Horticulture and Plant Protection, Inner Mongolia Agricultural University, Hohhot, China; ^2^Institute of Agro-Food Technology, Jilin Academy of Agricultural Sciences, Changchun, China; ^3^Jilin Provincial Key Laboratory of Agricultural Biotechnology, Jilin Academy of Agricultural Sciences, Changchun, China

**Keywords:** bacterial endophytes, Q2H1, potato, plant growth-promoting, whole-genome sequencing

## Abstract

**Introduction:**

Endophytes are non-pathogenic inhabitants of healthy plant tissues and have been found to promote plant growth and health. The endophytic bacterial strain Q2H1 was isolated from the roots of the potato and was identified to exhibit growth-promoting effects in potato plants.

**Methods:**

Whole-genome sequencing was performed to reveal the mechanism underlying its growth-promoting effect. The obtained sequencing data of approximately 5.65 MB encompassed 5,533 coding sequences. Of note, nine secondary metabolite gene clusters, including siderophore gene clusters, closely associated with plant growth promotion (PGP) were predicted by antiSMASH software. Comparative genomic analysis revealed that Q2H1 belongs to the genus *Peribacillus*. By gene function annotation, those genes related to plant growth-promoting activities, including indole-3-acetic acid (IAA) synthesis in tryptophan metabolism, siderophore biosynthetic activity, phosphate solubilization, nitrogen fixation, and related genes, were summarized. IAA (14.4 μg/ml) was presumptively produced by Q2H1 using the Salkowski colorimetric method. A total of five genes, namely, *phoU, pstB, pstA1, pstC*, and *pstS*, were annotated for phosphate solubilization, which is associated with the ability of the Q2H1 strain to solubilize phosphate under *in vitro* conditions.

**Results:**

It is revealed that genes in the Q2H1 genome associated with nitrogen fixation belonged to three groups, namely, nitrogen fixation (*nifU, sufU, salA*, and *nifS*), nitrogen metabolism (*nirA, nrtB*, and *nasA*), and glutamate synthesis (*glnA, gltB, gltD*, and *gudB*), supported by evidence that Q2H1 grew on medium without nitrogen. We have also identified a siderophore gene cluster located on the chromosome of Q2H1, including seven genes (*viz., rbsR, rhbf, rhbE, rhbD, rhbC, rhbA, ddc*, and an unknown gene). In the *in vitro* assay, a prominent brown circle around the colony was produced on the chrome azurol S medium at 48 and 72 h post-inoculation, indicating that the siderophore gene cluster in Q2H1 harbored the ability to produce siderophores.

**Conclusion:**

In summary, these findings implied that identifying strain-specific genes for their metabolic pathways in bacterial endophytes may reveal a variety of significant functions of plant growth-promoting mechanisms.

## Introduction

Endophytes are microbes that live in plants but do not cause plant diseases (Gouda et al., 2016). Cavalcante and Dobereiner (1988) first identified and isolated an endophytic nitrogen-fixing bacterium from sugarcane tissue and named it *Acetobacter diazotrophicus* because of its ability to oxidize ethanol to acetic acid and fix nitrogen under acidic conditions. Endophytes have been demonstrated to play crucial roles in plant disease resistance, secondary metabolite synthesis, plant growth promotion (PGP), and environmental stress resistance (Patel and Archana, 2017; Afzal et al., 2019; Ouyabe et al., 2020; Lopes Antunes et al., 2022).

Several studies have shown that endophytes regulate plant growth by nitrogen fixation, phosphate solubilization, siderophore production, 1-aminocyclopropane-1-carboxylate (ACC) deaminase activity, and indole-3-acetic acid (IAA) synthesis (Yan et al., 2018). Endophytic bacteria from rice plants (*Oryza sativa*) were the earliest investigated plant endophytes, which showed versatile PGP activities, such as phosphate solubilization, siderophore production, and IAA synthesis (Etesami et al., 2014). Endophytes and symbiotic nitrogen-fixing bacteria in the nodules of leguminous plants have been demonstrated to improve the pathogen resistance of host plants (Zgadzaj et al., 2015). In sugarcane, endophytic bacteria, including *Enterobacter* sp. and *Klebsiella* sp., were found to significantly promote host plant growth by nitrogen fixation (Lin et al., 2015; Taule et al., 2016), which was also reported to assist in stubborn plant growth on barren dunes (Zhang and White, 2021). *Jatropha curcas* L., a fuel plant that grows on barren sandy soil, contains a rich population of growth-promoting and nitrogen-fixing bacteria (*Methylobacterium* strain L2-4), which significantly increases host plant growth and seed yield (Madhaiyan et al., 2014). Endophytes isolated from potato roots exhibited the characteristics of nitrogen fixation, phytohormone production, and resistance against pathogenic bacteria. It is also demonstrated that inoculation of endophytic bacteria in potatoes increased the shoot and root biomass of the plant and reduced the pathogenic bacterial biomass (Pageni et al., 2014).

Endophytes colonize plants, stably protecting them from environmental stresses; however, they independently divide, reproduce, and transmit inside plants (Adeleke et al., 2021). *Azorhizobium caulinodans* is known to enter the root system of cereals *via* intercellular invasion between epidermal cells and to internally colonize the plant, including the xylem (Dreyfus et al., 1988). Many endophytic bacteria have been reported to successfully colonize non-leguminous plants, such as rice, wheat, maize, and sugarcane, forming symbiotic relationships and fixing nitrogen (Santoyo et al., 2016). It was reported that plant height, fresh weight, and chlorophyll content of sugarcane significantly increased when inoculated with the endophytic nitrogen-fixing bacteria *Klebsiella* sp. DX120E was observed by GFP-labeled DX120E, colonization in root hairs, new lateral roots, wounds, and leaves (Gu et al., 2018). The colonization of endophytic nitrogen-fixing bacteria in plants has an obvious advantage to plant growth over rhizosphere and symbiotic nitrogen-fixing bacteria.

In most cases, the mechanism of PGP by endophytes may result from either better uptake of soil nutrients or endophytic nitrogen fixation (Santoyo et al., 2016). Furthermore, endophytes can also secrete phytohormones such as IAA, gibberellin, and cytokinin that stimulate plant growth (Orozco-Mosqueda et al., 2020). IAA synthesis plays a vital role in promoting plant growth. *Bacillus subtilis* LK14 was reported to produce IAA and ACC deaminase *in vitro*. After the inoculation of endophytic *Bacillus subtilis* LK14 in tomato plants, the biomass of the shoot and root and the chlorophyll content significantly increased (Khan et al., 2016). Various IAA biosynthetic pathways have been proposed for endophytes, including tryptophan-dependent and tryptophan-independent pathways. Tryptophan-dependent pathways include indole-3-pyruvate (IPyA), indole acetamide synthesis (IAM), tryptamine (TAM), indole-3-acetonitrile (IAN), and tryptophan side chain oxidase (TSO) pathways (Liu et al., 2008). The TAM pathway is extensively studied in bacteria. A previous study found that *Azospirillum* can convert exogenous tryptamine into IAA (Normanly, 2010). It was reported that *Pantoea agglomerans* contain both IAM and IPyA genes to promote IAA synthesis. Also, the bacterial pathogen *Pseudomonas syringae* is reported to catalyze the formation of IAA from indole-3-acetaldehyde (IAAld) by indole-3-acetaldehyde dehydrogenase (AldA) (Zhao, 2012).

Siderophores are produced by endophytes. They usually combine with iron to form complexes, contributing to iron uptake directly from the complexes or by exchanging ligands (Garzón-Posse et al., 2019). With the increase in iron uptake from bacterial siderophores, plants increase their development and growth, for example, in rice, wheat, corn, and other crops (Santoyo et al., 2016; Afzal et al., 2019). Siderophores can form steady complexes with heavy metals, such as Cd, Cu, Pb, Zn, or radionuclide-U, reducing phytotoxicity in heavy metal-contaminated areas and improving plant growth (Grobelak and Hiller, 2017).

In addition to promoting plant growth, endophytes enhance the tolerance of host plants to abiotic and biotic stresses. Compared with the control, inoculation with the endophyte *Neotyphodium* spp. enhanced the tolerance of grasses to drought stress (Malinowski and Belesky, 2000). Inoculation with the bacterial endophyte *Burkholderia phytofirmans* strain PsJN can promote the growth of grapes, maintain physiological activity at low temperatures, and improve its ability to resist cold stress (Redman et al., 2002; Barka et al., 2006). Endophytes also play a significant role in salt stress and phytoremediation of contaminated soil and water (Mei and Flinn, 2010). Endophytes can also decrease biotic stress in plants. An isolate of *Burkholderia* sp. KJ006 from rice soil has a broad spectrum of antifungal properties (Cho et al., 2007). *Bacillus subtilis* (Lu144) significantly reduced the incidence of bacterial wilt in mulberry before infection by the pathogen. In tomato plants infected with potato virus Y (PVY), potato virus X (PVX), endophytic *Bacillus subtilis* 26D, and Ttl2 were reported to induce systemic resistance (ISR) by activating the transcription of salicylic acid- and jasmonic acid-related genes and regulating hormone levels, which resulted in reduced virus accumulation in plants (Veselova et al., 2022).

Whole-genome analysis of endophytes can be used to classify genes associated with PGP activities while providing insights into their molecular and functional mechanisms (Kamada et al., 2015; Lo et al., 2018; Lin et al., 2019; Franco-Sierra et al., 2020; Guo et al., 2020). Whole-genome sequencing of the *Enterobacter roggenkampii* ED5 strain, an endophytic strain of sugarcane, revealed a series of genes associated with PGP activity, including nitrogen fixation, production of plant hormones, biotic and abiotic stresses, induction of resistance, and root colonization (Guo et al., 2020). Based on the whole-genome sequencing data of *Bacillus subtilis* EA-CB0575, possible mechanisms of PGP were proposed by analyzing metabolic pathways (Franco-Sierra et al., 2020). Whole-genome sequencing of endophytic *Bacillus toyonensis* BAC3151 revealed its potential role in anti-microbial development associated with controlling crop diseases after analyzing secondary metabolites (Lopes et al., 2017).

In our previous study, we isolated a bacterial strain Q2H1 from potato roots treated at high temperatures and reported that it had an apparent growth-promoting effect on the potato; however, its underlying mechanism was unknown. In the current study, we determined the PGP activity of Q2H1 in potatoes and explored the possible PGP mechanisms of Q2H1 by whole-genome sequencing analysis. We analyzed the genomic characteristics of Q2H1 and compared them with those of other species. Gene clusters that were possibly related to IAA production, siderophore production, phosphate solubilization, and nitrogen fixation were analyzed. Our findings investigated the fundamental knowledge of endophytic strain Q2H1 and possible mechanisms of PGP, which will be helpful for its potential future application in enhancing potato production.

## Materials and methods

### Q2H1 strain and culture conditions

The endophytic strain Q2H1 was isolated from potato (Atlantic) roots and stored at –80°C with glycerol (50%). The cells were cultured in Luria–Bertani (LB) medium (tryptone: 10 g, yeast extract: 5 g, NaCl: 10 g, agar: 15 g in 1,000 ml of distilled water) at 28°C in an electrothermal constant-temperature incubator.

Genomic DNA was extracted from a 12-h logarithmic growth period LB liquid cell suspension of endophytic strain Q2H1 using a bacterial genomic DNA extraction kit (Tiangen Biotech, Beijing, China). DNA quality and concentration were estimated using NanoDrop One (Thermo Scientific). The 16S rRNA gene of Q2H1 was amplified by polymerase chain reaction (PCR) using a pair of primers (7F and 1540R). PCR amplification was performed using genomic DNA as the template (Supplementary Table 1). The PCR product was purified and sequenced by BGI (Shenzhen, China). The sequencing results were compared with the National Center for Biotechnology Information (NCBI) GenBank database. A phylogenetic tree was created using Molecular Evolutionary Genetic Analysis (MEGA, version 7.0, Mega Limited, Auckland, New Zealand).

### Plant growth-promoting assay of Q2H1 in potatoes in a greenhouse

The Q2H1 strain was stored at –80°C, and the inoculation loop was dipped in the bacterial solution and streaked onto a newly prepared LB solid medium plate at 28°C until the growth of the first colony. Q2H1 colonies were picked using an inoculation loop and then were inoculated into 100 ml of liquid LB medium at 28°C for 24 h at 180 rpm. The resultant liquid was then centrifuged at 4,000 rpm for 10 min, and an equal volume of sterile water was used to suspend, dilute to OD_600_ = 1, and then set aside. For this study, the Atlantic potato variety was used. The soil and nutrient soil were mixed in a 1:1 ratio by volume, watered, and mixed thoroughly. The exact size of potato sprouting tubers was sown in pots, with one piece each. When four or more leaves were grown from the tubers, 30 ml of the aforementioned bacterial suspension was inoculated with root irrigation, and 30 ml of sterile water was added as the negative control. The plants were grown under greenhouse conditions (22°C, 16-h light, and 8-h dark). To examine the effects of Q2H1 on potato growth, plant height, fresh weight, root weight, and chlorophyll content were measured 30 days post-inoculation. The experiment was repeated three times.

### Sample preparation of Q2H1 and genome sequencing

A single colony of Q2H1 from the culture plate was inoculated into 20 ml of liquid LB medium and cultured at 28°C with shaking at 180 rpm for 12 h. Bacterial liquid (2 ml) was taken in a centrifuge tube and centrifuged at 14,000 × *g* for 1 min at 25°C; the medium was discarded; and the bacteria were quickly transferred to liquid nitrogen for 1–3 h and then transferred to –80°C for storage.

Q2H1 samples were sent to Biomarker Technologies (Beijing, China) for genome sequencing with PacBio platform single-molecule real-time (SMRT) sequencing. The experimental procedure was carried out following the standard protocol provided by PacBio and included sample quality detection, library construction, library quality detection, and library sequencing. PacBio sequencing technology uses an SMRT chip as a sequencing carrier. DNA polymerase was bound to the template in the nanopore inside the SMRT chip, and the dNTP mix was labeled with four-color fluorescence. In the base pairing stage, different bases labeled with different lights are determined according to the wavelength and peak value of the light. SMRT Link version 8.0 software was used to process the off-machine PB data to obtain circular consensus sequencing (CCS) data. After further filtering the reads of short fragments (length < 2,000 bp), the total dataset was obtained. Hifiasm v0.12 (r304) software was used to assemble the filtered CCS data, and Pilon version 1.22 software was used for further error correction using second-generation data to obtain genomes with higher accuracy for subsequent analysis.

### Genome component analysis and functional annotation

Genomic component analyses include coding gene prediction (Hyatt et al., 2010), repeat sequence prediction, clustered regularly interspaced palindromic repeats (CRISPR) sequence prediction, pseudogene prediction, gene island prediction, prophage prediction, gene cluster prediction, promoter prediction, and paralogous gene prediction (Bland et al., 2007; Bertelli and Brinkman, 2018; Blin et al., 2019). Biosynthetic gene clusters (BGCs) in bacterial genome sequences were identified and analyzed using antiSMASH v5.0.0 software and combined with the results of NCBI BLAST alignment analysis. The sequencing results were compared with seven databases, namely, the non-redundant protein database (Nr), gene ontology (GO), Kyoto Encyclopedia of Genes and Genomes (KEGG), eggNOG, Pfam, SwissProt, and TrEMBL for gene function annotation and the annotation information of each database. Using the predicted genomic data, a genome map of Q2H1 was created using Circos v0.66 software (Krzywinski et al., 2009). From the KEGG database, the genes related to IAA production, phosphate solubilization, and nitrogen fixation were analyzed. Gene clusters from the secondary metabolites of Q2H1 were investigated.

### Comparative genomics analysis of Q2H1

Among average nucleotide identity (ANI) software calculations and tools, Jspecies is one of the most commonly used. We used the online website of JspeciesWS^1^ to determine whether two or more genomes belonged to the same species by calculating the ANI value (Richter et al., 2016). The genomic data of eight closely related strains were downloaded from the NCBI database. ANI results were analyzed using TBtools software (version v1.098765), and the results were displayed as heat maps (Chen et al., 2020).

Based on the ANI analysis results of the Q2H1 genome, two strains (*Peribacillus frigoritolerans* strain Ant232 and *Peribacillus simplex* strain NBRC 15720) with the closest genetic relationship to Q2H1 were used for comparative genomic analysis. The genome sequences of *P. frigoritolerans* strain Ant232 (GenBank accession number: CP084539.1) and *P. simplex* strain NBRC 15720 (GenBank accession number: CP017704.1) were downloaded from the NCBI database. To conduct the gene family clustering analysis, OrthoMCL version 2.0 software (Li et al., 2003) was used to perform family clustering on the protein sequences predicted by the sequencing strains and the protein sequences of the reference genome. We then analyzed the gene families, including strain-specific gene families and common gene families, in different strains. Venn diagrams or petal diagrams were constructed to identify gene families. Functional annotations based on the Pfam database were used in this study. For genome collinearity analysis using the Q2H1 genome as the reference genome, the protein sequences of Q2H1 were compared with those of the reference genome using BLAST (Wang et al., 2012). Then, according to the position of the homologous gene in the genome sequence, a collinear relationship was obtained at the nucleotide level.

### Detection of siderophore from Q2H1 by chrome azurol S assay

To confirm the expression of the siderophore gene cluster, we performed an *in vitro* assay in chrome azurol S (CAS) assay medium (sucrose: 2 g, casein acid hydrolyzed: 3 g, 1 mM CaCl_2_: 1 ml, 1 mM MgSO_4_⋅7H_2_O: 20 ml, agar: 20 g, distilled water: 1,000 ml. The mixture was sterilized in an autoclave at 121°C for 30 min) and waited until the medium temperature dropped approximately to 60°C. Subsequently, 5 ml of phosphate buffer and 5 ml of CAS staining solution were added per 100 ml of medium. The CAS staining solution was dissolved in water to form a blue complex with hexadecyl trimethyl ammonium bromide (CTAB) (Chen, 2020). To culture Q2H1 in LB solid medium, a single colony was picked with a sterile toothpick and inoculated on the CAS detection medium. When the complex was degraded, it produced a yellow or brown halo around the colony. The experiment was repeated three times, the aperture diameter was measured at 48 and 72 h, and the siderophore ability was analyzed.

### Nitrogen-fixing and phosphate solubilization of Q2H1

Phosphate solubilization experiments were validated on organic phosphate medium [glucose: 10.0 g, (NH_4_)_2_SO_4_: 0.5 g, NaCl: 0.3 g, KCl: 0.3 g, FeSO_4_⋅7H_2_O: 0.03 g, MgSO_4_⋅7H_2_O: 0.3 g, MnSO_4_⋅4H_2_O: 0.03 g, Ca_3_(PO_4_)_2_: 5 g, agar: 15 g, distilled water: 1,000 ml, pH 7.0] and inorganic phosphate medium (NaCl: 5.0 g, beef extract: 5.0 g, peptone: 10.0 g, agar: 15 g, distilled water: 1,000 ml, pH 7–7.5, add 3 ml of fresh egg yolk liquid to each 50 ml for temporary use) (Gao et al., 2015). A single colony of Q2H1 was picked using a sterile toothpick and inoculated to the inorganic phosphate medium and organic phosphate medium. The cells were incubated at 28°C for 7 days to observe the development of a halo. Nitrogen-fixing ability was measured using Ashby nitrogen-free medium (mannitol: 10 g, KH_2_PO_4_: 0.2 g, MgSO_4_⋅7H_2_O: 0.2 g, NaCl: 0.2 g, CaSO_4_⋅2H_2_O: 0.1 g, CaCO_3_: 5 g, agar: 15 g, distilled water: 1,000 ml) (Singh et al., 2020). Q2H1 was inoculated into the Ashby medium at 28°C for 7 days to determine its viability.

### Indole-3-acetic acid production assay

Indole-3-acetic acid production was estimated using the Salkowski colorimetric method in the presence of tryptophan (Patten and Glick, 2002). Strains were grown overnight in the DF medium (Zhang et al., 2016), and 200 μl was transferred to the DF medium containing 0.1% *L*-tryptophan. The strains were cultured at 28°C for 7 days and then centrifuged at 8,000 rpm for 10 min. In total, 1 ml of the fermentation supernatant was mixed with 2 ml of Fe-H_2_SO_4_ solution (1 ml of 0.5 M FeCl_3_⋅6H_2_O in 75 ml of 6.13 M H_2_SO_4_) and placed in a dark room for 45 min. The IAA concentration was presumed by measuring the absorbance of the samples at 450 nm.

### Antagonism assay against phytopathogenic fungi

In the *in vitro* antifungal activity test of the strain Q2H1, five pathogenic fungi, namely *Fusarium oxysporum*, *Fusarium commune*, *Fusarium graminearum*, *Rhizoctonia solani*, and *Stemphylium solani*, were tested on the PDA medium. These pathogenic fungi are from the College of Horticulture and Plant Protection, Inner Mongolia Agricultural University, China. A 5 mm diameter of the culture medium from each pathogenic fungi was cut and placed on the PDA plate together with the Q2H1 strain. The plate containing only pathogenic fungi was used as the control. Then, the plates were incubated at 28°C for 3–5 days, the fungus radii were measured, and the antifungal rates were calculated.

### Statistical analysis

All genomic analyses were performed following the manufacturer’s instructions. All PGP trials were performed in triplicate, and data were analyzed using analysis of variance and Duncan’s multiple range test.

## Results

### Plant growth promotion of endophytic strain Q2H1 in potato

**FIGURE 1:**
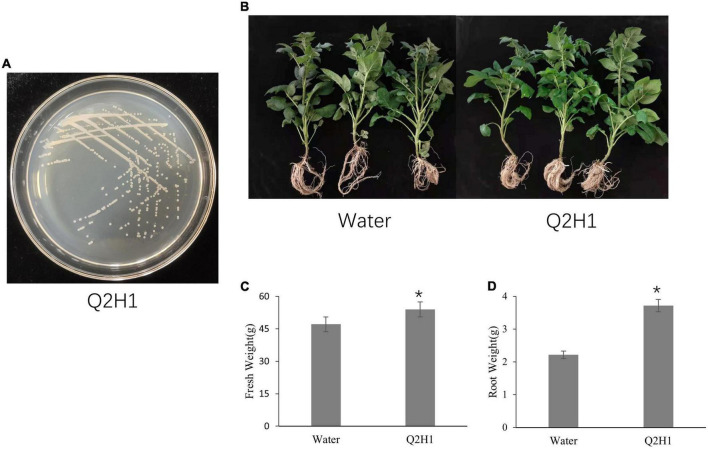
Plant growth promotion of endophytic strain Q2H1 in potato: **(A)** Colony morphology of strain Q2H1 on LB medium; **(B)** photographs of potato plants at 30 days post-inoculation with Q2H1; **(C,D)** fresh weight and root weight of potato plants measured at 30 days post-inoculation with Q2H1; *show the significant differences (*P* < 0.05) according to multiple ranges of LSD.

We isolated the endophytic strain Q2H1 from potato roots (Figure 1A). The Q2H1 growth promotion assay was performed on potato plants grown in a greenhouse (Figure 1B). Compared with the control (treated with sterile water), potato plants treated with Q2H1 bacterial suspension showed an average increase in fresh weight of 6.83 g (Figure 1C) and an average increase in root weight of 1.5 g (Figure 1D), with significant differences. Plant height increased by 1.11 cm, chlorophyll content increased by 3.89, and the difference was not significant compared with the control.

### Genomic properties of endophytic strain Q2H1

The Q2H1 strain was identified by 16S rRNA gene sequencing, and the obtained sequences were matched with the nucleotide sequences of the National Center for Biotechnology Information (NCBI) GenBank database using the Basic Local Alignment Search Tool (BlastN) program. We constructed a phylogenetic tree using the BLAST results for Q2H1, and the results are shown in Supplementary Figure 1. Q2H1 was preliminarily identified as *Peribacillus.*

**FIGURE 2:**
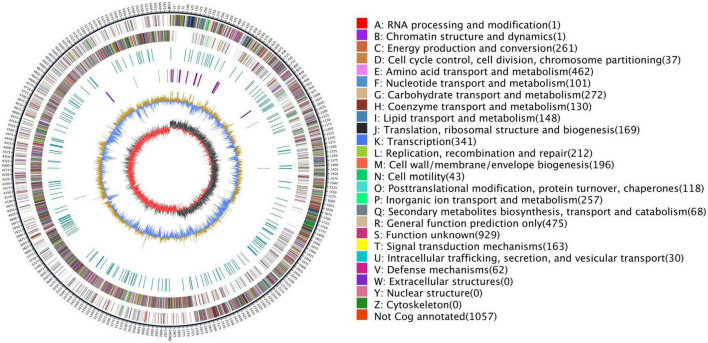
Genome map of Q2H1: The outermost circle is the indication of the genome size; the second and third circles are genes on the positive and negative strands of the genome, respectively; and different colors represent different COG functional classifications; the fourth circle is the repeat sequence; the fifth circle is tRNA and rRNA, blue is tRNA, and purple is rRNA; the sixth circle is the GC content; and the innermost circle is GC skew. A–Z, respectively, show the functional classification of the CDS genes in the chromosome.

To explore the genomic properties of the endophytic strain Q2H1, we performed whole-genome sequencing using single-molecule real-time (SMRT) sequencing (Biomarker Technologies Beijing China). The general characteristics of the Q2H1 genome are listed in Table 1. The Q2H1 genome contains a chromosome of 5,703,943 bp and three plasmids with an average genome-wide GC content of 40.28%. The genome was predicted to contain 5,533 coding sequences (CDSs). Using the CRT, antiSMASH, and IslandPath-DIMOB software, we predicted four clustered regularly interspaced palindromic repeat (CRISPR) sequences, nine gene clusters, and four gene islands. Moreover, the genome of Q2H1 includes 90 tRNA and 40 rRNA genes (13 5S rRNA, 14 16S rRNA, and 13 23S rRNA) (Kalvari et al., 2018; Chan and Lowe, 2019). The number of CDSs assigned to the KEGG, eggNOG, and GO databases was 2,626, 4,475, and 4,039, respectively. The results of plasmid genome annotation are shown in Supplementary Table 2 and Supplementary Figure 2. Circos visualizes the genome, which could more clearly explore the relationship between genomic components or locations (Figure 2). The different colors in Figure 2 represent different functions of genes annotated in the COG database, including amino acid transport metabolism, general function prediction, secondary metabolism biosynthesis, transport, and catabolism. A complete genome sequence of this strain has been submitted at GenBank with accession numbers CP110132-CP110135.

**TABLE 1:** Genome characteristics of endophytic strain Q2H1.

Characteristics-Q2H1	Value
Genome size (bp)	5,781,076
GC content (%)	40.28
Topology	Circular
Chromosome size (bp)	5,703,943
Chromosome	1
Plasmid	3
tRNA	90
rRNA (5S, 16S, 23S)	40
Protein-coding genes (CDS)	5,533
Genomic islands	4
CRISPR	4
Gene cluster	9
Genes assigned to NR	5,395
Genes assigned to GO	4,039
Genes assigned to KEGG	2,626
Genes assigned to eggNOG	4,475
Genes assigned to Pfam	4,578
Genes assigned to Swiss-Prot	3,319
Genes assigned to TrEMBL	3,319

### Comparative genomics analysis of Q2H1 strain

**FIGURE 3:**
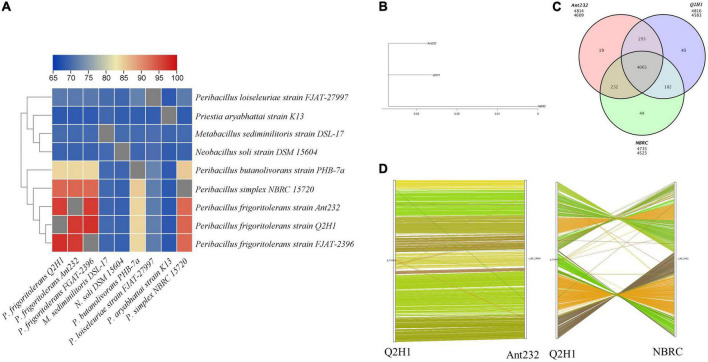
Comparative genomics analysis of Q2H1 strain: **(A)** Heat maps of average nucleotide identity (ANI) between strain Q2H1 and phylogenetically eight closely related species. **(B)** Evolutionary relationship between Q2H1 and two species. **(C)** Venn diagram of gene family statistics. **(D)** Genome collinearity of Q2H1/Ant232 and Q2H1/NBRC.

To analyze the similarity of the Q2H1 strain with other closely related species, phylogenetic and comparative genomic analyses were performed. Heat maps of average nucleotide identity (ANI) between the strain Q2H1 and the eight closest phylogenetically related species were created by TBtools software. ANI analysis showed that the ANI similarity between the Q2H1 genome and *P. frigoritolerans* strain FJAT-2396 was 97.34% and that between Q2H1 genome and *P. frigoritolerans* strain Ant232 was 96.53%. Genome similarity between the Q2H1 genome and *P. simplex* NBRC 15720 was 93.08%. These ANI results indicated that Q2H1 belonged to *P. frigoritolerans* (Figure 3A).

Analysis of the 16S rRNA gene and ANI showed that *P. frigoritolerans* strain FJAT-2396 was most similar to Q2H1, but the NCBI genome database retrieved only one scaffold for the *P. frigoritolerans* strain FJAT-2396 genome, whereas *P. frigoritolerans* strain Ant232 (named as Ant232) and *P. simplex* strain NBRC 15720 (named as NBRC) had complete genome information. Therefore, Ant232 and NBRC were selected for comparative genomic analysis. To analyze the difference between the Q2H1 strain and the selected Ant232 and NBRC strains, a comparative genomics analysis was performed. An evolutionary tree showing the evolutionary relationship between species was created by PhyML software (Guindon et al., 2010; Figure 3B). These three strains belonged to the same clade, and the evolutionary relationship between Q2H1 and Ant232 was closer than that between Q2H1 and NBRC. Q2H1 is most similar to the *P. frigoritolerans* strain Ant232. Gene family cluster analysis revealed 4,583 gene families in the Q2H1 strain, 4,609 in the Ant232 strain, and 4,523 in the NBRC strain. The number of gene families shared by the three strains was 4,065, and 43 unique genes were identified in the Q2H1 strain, 19 in the Ant232 strain, and 44 in the NBRC strain (Figure 3C).

To analyze the positional and evolutionary relationships of homologous genes on chromosomes between Q2H1 and closely related species, genome collinearity analysis was performed to compare the protein sequence of Q2H1 with the protein sequence of each reference genome using BLAST. We then obtained collinearity at the nucleic acid level according to the position information of the homologous gene on the genome sequence. The results showed that Q2H1 and Ant232 had good collinearity at the nucleotide level, and only translocations of the individual genes occurred (Figure 3D). However, in the collinear map of Q2H1 and NBRC, it can be seen that there is only a localized region of collinearity, and a large number of gene inversion, translocation, and genome rearrangement events occur in NBRC. The accumulated variation between Q2H1 and Ant232 genomes was lower. More features were retained from the ancestors, and the order of the genes was relatively consistent, indicating that the two have a shorter differentiation time. The difference between Q2H1 and NBRC may be due to the longer differentiation time, which leads to the accumulation of inter-species variation, the reduction of shared features, and the acquisition of fewer collinear fragments.

### Predictive gene clusters involved in the synthesis of secondary metabolites in Q2H1

To identify the gene clusters involved in the synthesis of secondary metabolites in Q2H1, antiSMASH software prediction and NCBI BLAST comparison analyses were performed. The results showed that the Q2H1 strain encodes nine gene clusters involved in secondary metabolite synthesis. Among them, there were three non-ribosomal peptide synthases (NRPSs), including NRPS-type koranimine, meilingmycin, SF2575, one betalactone (betalactone-type fengycin), one type III PKS (polyketide synthase), two terpenes, one linear azol(in)e-containing peptides (LAPs), and one siderophore cluster (Table 2). In comparison to the known gene clusters, the similarity with the koranimine-encoded gene cluster reached 87%, and the similarity of the fengycin-encoded gene cluster was 40%. However, the similarity of the other two, meilingmycin and SF2575, is quite low (2 and 4%, respectively), indicating that some secondary metabolites may be specific to Q2H1. In addition, five unknown metabolites were encoded, which were initially predicted to be T3PKS, terpene, LAP RiPP-like, terpene, and siderophore, which requires further study.

**TABLE 2:** Predictive gene clusters involved in synthesis of secondary metabolites in Q2H1.

Region of genome		Most similar known cluster			
From	To	Type	Productions	Similarity	Resources
724,080	783,280	NRPS	Koranimine	87%	*Bacillus* sp. NK2003 (Evans et al., 2011)
944,230	987,127	NRPS	Meilingmycin	2%	*Streptomyces nanchangensis* (He et al., 2010)
2,494,140	2,518,309	Betalactone	Fengycin	40%	*Bacillus velezensis* FZB42 (Koumoutsi et al., 2004)
5,216,386	5,268,892	NRPS, NRPS-like	SF2575	4%	*Streptomyces* sp. SF2575 (Pickens et al., 2009)
3,456,490	3,497,578	T3PKS	–		
3,634,729	3,654,767	Terpene	–		
4,577,196	4,600,731	LAP, RiPP-like	–		
5,298,831	5,320,726	Terpene	–		
5,647,469	5,662,981	Siderophore	–		

### Expression of plant growth-promoting traits found in Q2H1 genome *in vitro*

Through whole-genome sequencing, we found a series of genes possibly related to growth promotion, IAA production (Supplementary Figures 3, 4), phosphate solubilization (Supplementary Figure 5), siderophore production, and nitrogen fixation (Supplementary Figure 6) in the Q2H1 genome. This helped us better understand the PGP mechanisms of Q2H1.

The key IAA-producing gene *iaaM* and a series of related genes were found in the genome, which underlined the IAA-producing ability of Q2H1. Tryptophan is a precursor of bacterial IAA synthesis. We identified the tryptamine pathway (TAM pathway) in the tryptophan metabolism KEGG pathway map (Supplementary Figure 3). In addition, a set of tryptophan biosynthesis genes, *trpABCDEFGDPS*, was found in the tryptophan biosynthesis pathway map (Supplementary Figure 4) and was closely related to IAA synthesis (Guo et al., 2020; Table 3). The Salkowski colorimetric method was used for the *in vitro* presumptive determination of IAA, and Q2H1 produced IAA (14.4 μg/ml).

**TABLE 3:** Predicted genes associated with PGP in Q2H1 genome.

PGP activities description	Gene name	Gene annotation	Chromosome location
IAA production	*iaaM*	Tryptophan 2-monooxygenase	2496986-2498470 (GE002328)
	*trpA*	Tryptophan synthase alpha chain	1606527-1607306 (GE001489)
	*trpB*	Tryptophan synthase beta chain	1605329-1606534 (GE001488)
	*trpC*	Indole-3-glycerol phosphate synthase	1603923-1604699 (GE001486)
	*trpD*	Anthranilate phosphoribosyltransferase	1602901-1603923 (GE001485)
	*trpE*	Anthranilate synthase component 1	1600877-1602271 (GE001483)
	*trpF*	*N*-(5’-phosphoribosyl)anthranilate isomerase	1604710-1605324 (GE001487)
	*trpGD*	Bifunctional protein TrpGD	1602268-1602873 (GE001484)
	*trpP*	Probable tryptophan transport protein	1292173-1292691 (GE001178)
	*trpS*	Tryptophan–tRNA ligase	1381331-1382317 (GE001264)
Phosphate metabolism	*phoU*	Phosphate-specific transport system accessory protein PhoU homolog	3738657-3739310 (GE003534)
	*pstB*	Phosphate import ATP-binding protein PstB	3739330-3740157 (GE003535)
	*pstA1*	Phosphate transport system permease protein PstA 1	3740173-3741051 (GE003536)
	*pstC*	Phosphate transport system permease protein PstC	3741055-3741993 (GE003537)
	*pstS*	Phosphate-binding protein PstS	3742093-3743058 (GE003538)
Nitrogen fixation	*nifU*	Involved in iron-sulfur cluster biogenesis	5205897-5206133 (GE005003)
	*sufU*	SUF system NifU family Fe-S cluster assembly protein	5262616-5263044 (GE005050)
	*salA*	Iron sulfur cluster binding proteins, NifH/frxC family	159243-160301 (GE000155)
	*nifS*	Putative cysteine desulfurase NifS	3943954-3945081 (GE003743)
Nitrogen metabolism	*nirA*	Ferredoxin–nitrite reductase	2856274-2857899 (GE002688)
	*nrtB*	Nitrate import permease protein NrtB	1426192-1426908 (GE001311)
	*nasA*	Nitrate transporter	2753712-2754917 (GE002596)
Glutamate synthesis	*glnA*	Glutamine synthetase	1879969-1881303 (GE001756)
	*gltB*	Glutamate synthase [NADPH] large chain	835311-839783 (GE000744)
	*gltD*	Glutamate synthase [NADPH] small chain	191089-192453 (GE000181)
	*gudB*	Cryptic catabolic NAD-specific glutamate dehydrogenase GudB	3451565-3452746 (GE003241)
Siderophore	rbsR	LacI family transcriptional regulator	5648068-5649054 (GE005416)
	-	Exodeoxyribonuclease v alpha	5650277-5651872 (GE005417)
	rhbF	IucA/IucC family siderophore biosynthesis protein	5652469-5654280 (GE005418)
	rhbE	Lysine 6-monooxygenase	5654302-5655630 (GE005419)
	rhbD	*N*-acetyltransferase	5655605-5656189 (GE005420)
	rhbC	IucA/IucC family siderophore biosynthesis protein	5656179-5657981 (GE005421)
	ddc	*L*-2,4-diaminobutyrate decarboxylase	5657984-5659492 (GE005422)
	rhbA	Diaminobutyrate–2-oxoglutarate aminotransferase	5659500-5660852 (GE005423)
Linear gramicidin synthase	*lgrB*	Linear gramicidin synthase subunit B	743660-751195 (GE000669)
	*lgrD*	Linear gramicidin synthase subunit D	751206-751472 (GE000670)
	*lgrC*	Linear gramicidin synthase subunit C	751499-756217 (GE000671)
	*lgrC*	Linear gramicidin synthase subunit C	756297-763976 (GE000672)
	*lgrC*	Linear gramicidin synthase subunit C	763973-766876 (GE000673)
	*lgrE*	Linear gramicidin dehydrogenase LgrE	766897-767628 (GE000674)

**FIGURE 4:**
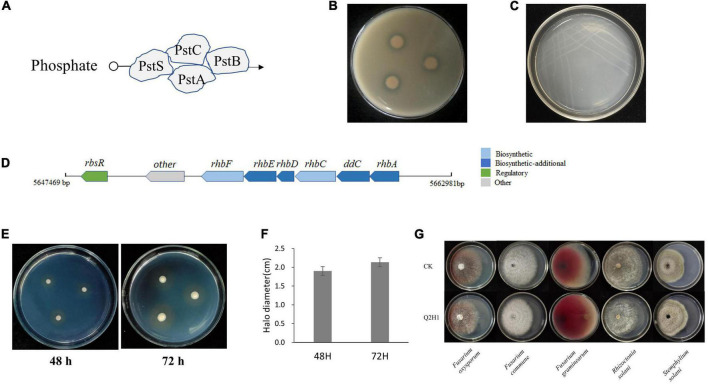
*In vitro* assay of PGP activity: **(A)** Phosphate transport pattern diagram. **(B)** Phosphate solubilization experiment in organic phosphate medium. **(C)** Ashby nitrogen-free medium. **(D)** Siderophore gene cluster arrangement. **(E)** Siderophore CAS detection medium. **(F)** Diameter of the halo in CAS medium at 48 h and 72 h. **(G)** Antagonism of Q2H1 against pathogenic fungi.

We also identified a cluster of genes associated with phosphate metabolism in the Q2H1 genome (Table 3 and Figure 4A). Through KEGG analysis, phosphate transporters encoded by *pstS*, *pstC*, *pstA*, and *pstB* were found in the phosphate metabolism pathway (Supplementary Figure 5). To determine whether these genes could be expressed *in vitro*, a phosphate solubilization assay was performed. The Q2H1 culture was dotted on organic and inorganic phosphate mediums grown for 7 days. The results showed obvious halos around the dots on the organic phosphate medium (Figure 4B). No halos were observed in the inorganic phosphate medium. This indicated that Q2H1 could efficiently dissolve organic phosphate in the medium.

We also identified a cluster of genes associated with nitrogen fixation in Q2H1 (Table 3). Unfortunately, we did not identify a complete nitrogenase gene cluster. Only *nifU*, *nifS*, and *sufU* genes were present in the strain genome. Annotation revealed that these genes were related to nitrogenase activity and were responsible for biological nitrogen fixation. The regulatory genes *nirA*, *nasA*, and *nrtB* are found in the nitrogen metabolism pathway. The protein encoded by *nrtB* transports extracellular nitrate into the cell, which is reduced to nitrate under the control of *nasA*, and nitrate produces ammonia under the action of *nirA* (Supplementary Figure 6). The glutamine synthase *gene glnA* and the glutamate synthase genes *gltB* and *gltD* were found in the nitrogen metabolism pathway map (Supplementary Figure 6). To test the nitrogen fixation ability of strain Q2H1, we performed an assay to grow Q2H1 in a medium without nitrogen. We found that Q2H1 could grow in a medium without nitrogen (Figure 4C).

We sorted the genes encoding siderophore clusters in the genome sequence of Q2H1. The siderophore synthesis pathway and related genes were also analyzed. The annotation results are shown in Table 3, which lays the foundation for an in-depth study of siderophores and reveals the mechanism of PGP. We found that the siderophore gene cluster was located on chromosome (5647469-5662981). A schematic representation of the siderophore gene cluster arrangement is shown in Figure 4D. To verify whether the siderophore gene cluster can be expressed *in vitro*, a siderophore test was carried out using the CAS detection medium. The results showed an apparent brown circle around the Q2H1 colony on the plate 48 h post-inoculation (Figure 4E). The halo expanded further after 72 h (Figure 4F), indicating that Q2H1 can produce siderophores.

However, we found the linear gramicidin synthase-related genes *lgrB*, *lgrC*, *lgrD*, and *lgrE* from SwissProt annotation, which may be associated with disease resistance (Table 3). According to the results of the antagonistic test of Q2H1 against five pathogenic fungi, we found that QH21 had no antagonistic activity (Figure 4G).

## Discussion

Bacteria in plant roots and rhizospheres benefit from root exudates. Some bacteria can enter plants as endophytes, which can establish a mutualistic association (Gaiero et al., 2013). It has been reported that the promotion of plant growth by endophytes may be due to nitrogen fixation (Lowman et al., 2016), phytohormone production, biological control of phytopathogens (through the production of antifungal or microbial agents), siderophore production, nutrient competition, induction of systemic acquired host resistance, or increasing availability of minerals (Ali et al., 2014; Santoyo et al., 2016; Mukherjee et al., 2017; Lata et al., 2018). Exploring the mechanisms of PGP will aid in the application of endophytic bacteria to enhance plant production.

In this study, we isolated an endophytic bacterial strain, Q2H1, from potato roots, which plays a growth-promoting role in potato plants. The fresh and root weights of potato plants were significantly increased. The average nucleotide identity (ANI) is an indicator for comparing the relationship between two genomes at the nucleotide level. ANI is defined as the average base similarity between the homologous fragments of two microbial genomes. It is characterized by a high degree of discrimination between closely related species, and it is generally considered that an ANI ≥ 95% indicates the same species (Goris et al., 2007; Yoon et al., 2017). Based on the 16S rRNA gene and ANI analysis, Q2H1 is closely related to *P. frigoritolerans*.

We performed whole-genome sequencing of Q2H1 to explore the possible mechanisms of PGP. Through comparative genome analysis, we found that evolutionary relationships suggest that these three strains belong to the same clade (Figure 3B); more specifically, Q2H1 and Ant232 are closer than Q2H1 and NBRC. Gene family cluster analysis of Q2H1 and both Ant232 and NBRC revealed that the three strains shared 4,065 genes, and Q2H1 had 43 unique genes. A considerable number of gene inversion, translocation, and genome rearrangement events occurred in NBRC, as can be seen in the collinear map of Q2H1 and NBRC. There was less variation in accumulation between the genomes of Q2H1 and Ant232. The fact that most ancestor-derived traits were retained and the gene order was largely constant suggests that the two strains took less time for differentiation. Good collinearity between the genomes of Q2H1 and Ant232 indicated relatively consistent gene sequences.

Next, we focused on the genes in the Q2H1 genome that were predictably associated with PGP. Studies have shown that secondary metabolites of plant endophytes can improve the resistance of plants to biotic and abiotic stresses (Gouda et al., 2016; Bharadwaj et al., 2020). A total of nine secondary metabolite gene clusters were predicted in the genome, including NRPS-type koranimine, meilingmycin, SF2575, betalactone-type fengycin, five unknown products, one type III PKS (polyketide synthase), two terpenes, one linear azol(in)e-containing peptides (LAPs), and one siderophore cluster (Koumoutsi et al., 2004; Pickens et al., 2009; He et al., 2010; Evans et al., 2011).

We found genes and enzymes involved in tryptophan metabolism in the KEGG metabolic pathway, which is closely related to the synthesis of indoleacetic acid. In addition, genes *iaaM*, *trpABCDEFPS*, and *trpGD* in the tryptophan synthesis pathway were also found. *iaaM* is a key gene in the IAM pathway, and *trpABCDFEPS* and *trpGD* are involved in tryptophan synthesis (Liu et al., 2008). This is consistent with the results of the IAA production assay *in vitro*. Similar to our findings, it was previously recognized that the presence of tryptophan-related genes in bacterial genomes is associated with IAA production, as described for *Enterobacter* strain 638 (Taghavi et al., 2010) and *Enterobacter cloacae* UW5 (Coulson and Patten, 2015). Asaf et al. (2018) identified a tryptophan biosynthesis gene (*trpABD*) involved in IAA production in *Sphingomonas* sp. LK11 genome.

A group of phosphate metabolism genes *phoU*, *pstA*, *pstB*, *pstC*, and *pstS* are presumed to have phosphate solubilization capabilities. Through functional annotation of Q2H1, we learned that these genes (*phoU*, *pstA*, *pstB*, *pstC*, and *pstS*) encode phosphate transporters, which together constitute the phosphate transport system. We also demonstrated that Q2H1 could solubilize organic phosphate *in vitro*. Similar to our results, other endophytes, such as *Bacillus subtilis* RS10 (Iqbal et al., 2021), *E. roggenkampii* ED5 (Guo et al., 2020), and *B. subtilis* EA-CB0575 (Franco-Sierra et al., 2020), have also been reported as phosphate solubilizers. Genes related to phosphate transport (*pstACS*) were found in the genome of *B. subtilis* strain RS10, which was confirmed *in vitro* and had favorable plant growth-promoting traits. These genes are also present in *E. roggenkampii* ED5 and have been confirmed *in vitro*.

Regarding nitrogen fixation, only individual nitrogen fixation-related genes *nifU*, *nifS*, *sufU*, and *salA* were found. The presence of *nifU* and *nifS*, which are required for the components of the enzymatic module encoding nitrogenase, was determined (Li et al., 2016). The *nifU* protein plays a major role in Fe-S cluster aggregation, which is necessary for nitrogen fixation (Smith et al., 2005). This suggests the possibility of the strain fixing environmental nitrogen, which was experimentally determined by *P. frigoritolerans* Q2H1 growth on the Ashby medium. Correspondingly, we found that Q2H1 was able to grow on a medium without nitrogen. The regulatory genes *nirA*, *nasA*, and *nrtB*, which can convert nitrate to ammonia, are found in the nitrogen metabolism pathway. Glutamine plays an important role in nitrogen metabolism and regulates nitrogenase activity in some nitrogen-fixing photosynthetic bacteria and *Azospirillum* spp. Glutamine was first shown to regulate nitrogenase activity in *Rhodospirillum rubrum* (Nordlund and Högbom, 2013). The glutamate synthesis-related genes *glnA*, *gltB*, *gltD*, and *gudB* were also annotated. The presence of *nifU*, *glnA*, and *gltBD* in the genome of the sugarcane endophyte *E. roggenkampii* ED5 confirms the nitrogen fixation effect of ED5 and can significantly promote sugarcane growth (Guo et al., 2020).

When plants grow in an iron-deficient environment, siderophore production by microorganisms can chelate Fe^3+^ ions that can be difficult for plants to absorb directly for its utilization. Siderophores are relatively low-molecular weight (500–1,000) organic chelators that bind to insoluble iron in the environment and form Fe^3+^–siderophore complexes. Siderophore-producing bacteria change the availability of iron in the soil through the chelation of siderophores, thereby improving the iron availability in the rhizosphere of plants and meeting the nutritional needs of iron for plant growth (Garzón-Posse et al., 2019). In this study, we found a siderophore gene cluster in the Q2H1 genome; however, the siderophore type was not determined. The siderophore gene cluster was evaluated and confirmed *in vitro*. Consistent with the present study, siderophore production has been detected in *Enterobacter cloacae* SBP8 (Singh et al., 2017) and *Pseudomonas* sp. UW4 (Duan et al., 2013), with multiple PGP properties.

## Conclusion

Using whole-genome sequencing, we obtained the sequence of *P. frigoritolerans* Q2H1. Several genes and gene clusters related to growth-promoting effects have also been identified. The PGP characteristics of Q2H1, including IAA production, phosphate solubilization, nitrogen fixation, and siderophore production, were annotated to genes in the genome, which were verified *in vitro*. Our results are important for investigating the mechanism of PGP and setting the groundwork for future plant growth applications.

## Data availability statement

The datasets presented in this study can be found in online repositories. The names of the repository/repositories and accession number(s) can be found in the article/Supplementary material.

## Author contributions

MZ, YW, QZ, and XY wrote the manuscript. MZ, HYZ, and HLZ designed the experiments. YW, ZS, YL, HH, and QZ performed the experiments. YW, DW, and BD analyzed the sequencing data. YZ, QZ, XY, and HYZ advised on the experimental design and English language editing. All authors read and agreed to the published version of the manuscript.

## Footnotes

1
http://jspecies.ribohost.com/jspeciesws/
