# Supplementary material for: Whole-genome analysis revealed the growth-promoting mechanism of endophytic bacterial strain Q2H1 in potato plants
Source: Front Microbiol. 2022 Dec 1;13:1035901. doi: 10.3389/fmicb.2022.1035901 (PMC9751815; doi:10.3389/fmicb.2022.1035901)
Supplement: Supplementary file 1 [file Data_Sheet_1.docx]

***Supplementary Material***

**Supplementary Tables**

**Supplementary Table 1.** 16S rRNA PCR amplification experiments conditions

| 16srRNA Gene PCR amplification experiments conditions | |
| --- | --- |
| Product size | About 1600bp |
| PCR Primer | 7F 5′-CAGAGTTTGATCCTGGCT-3′  1540R 5′-AGGAGGTGATCCAGCCGCA3′ |
| PCR conditions | Initial temperature (98℃ for 3m), start cycles (29), Denaturation (98℃ for 10s), Annealing (50℃ for 30s), Elongation (72℃ for 1m50s), Final extensions (72℃ for 10m). |

**Supplementary Table 2.** Plasmid annotation table

|  | Plasmid 1 | Plasmid 2 | | Plasmid 3 | |  |
| --- | --- | --- | --- | --- | --- | --- |
| Genome size (bp) | 39093 | | 26651 | | 11389 | |
| Topology | linear | | linear | | linear | |
| Protein-coding genes (CDS) | 29 | | 25 | | 18 | |
| GC content (%) | 31% | | 30% | | 33% | |

**Supplementary Figures**

**
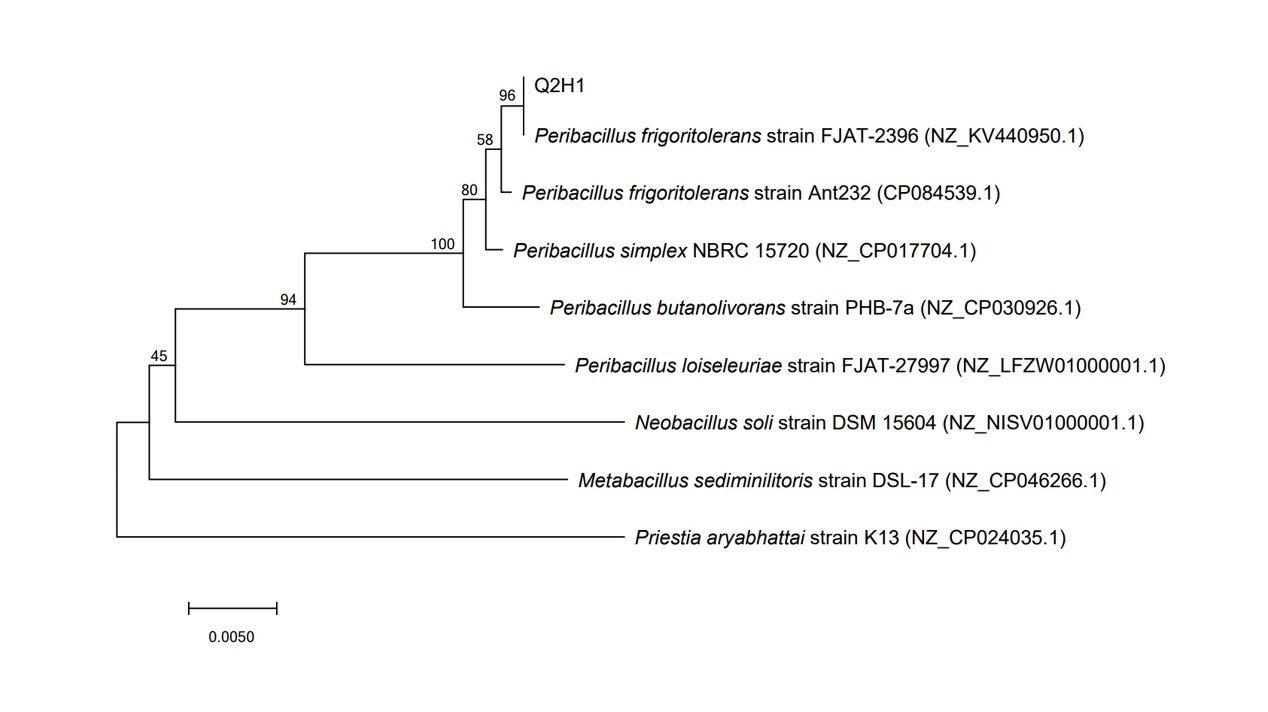
**

**Supplementary Figure 1.** Phylogenetic tree based on16S rRNA gene sequences. The evolutionary history was inferred using the Neighbor-Joining method.

**
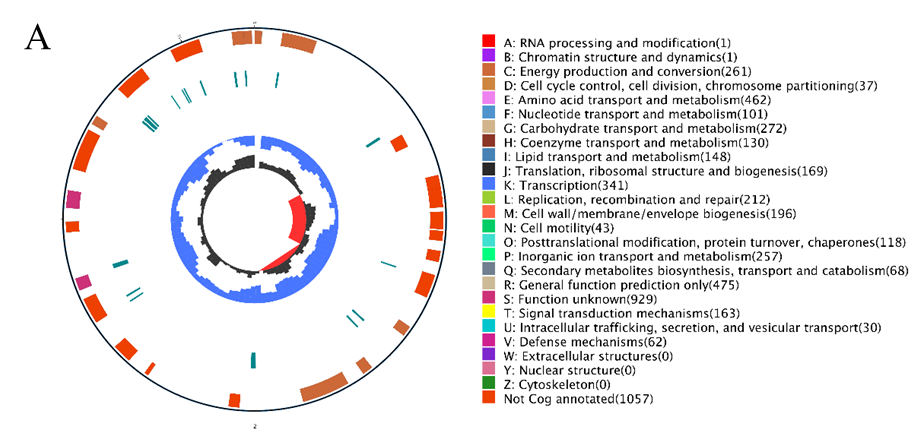
**

**
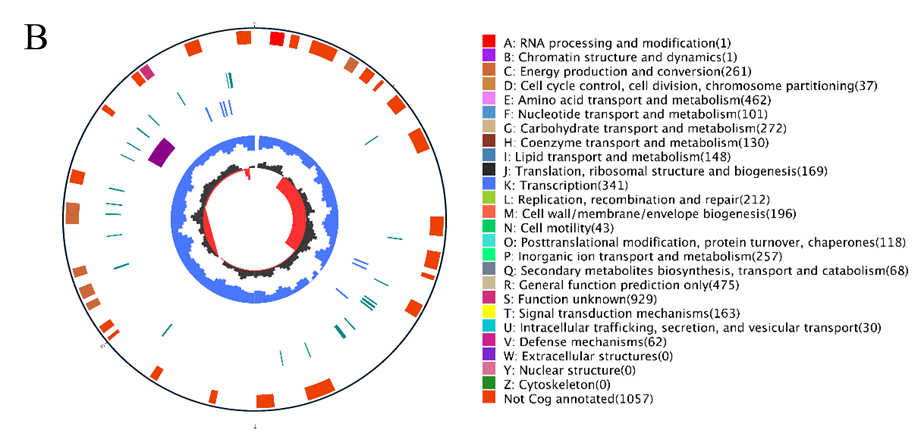
**

**
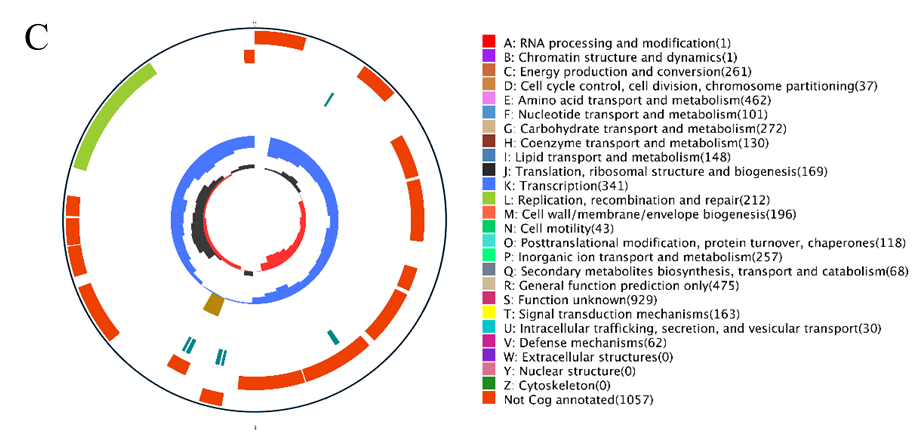
**

**Supplementary** **Figure 2.** The plasmid map of Q2H1 (**A**) plasmid 1 (**B**) plasmid 2 (**C**) plasmid 3


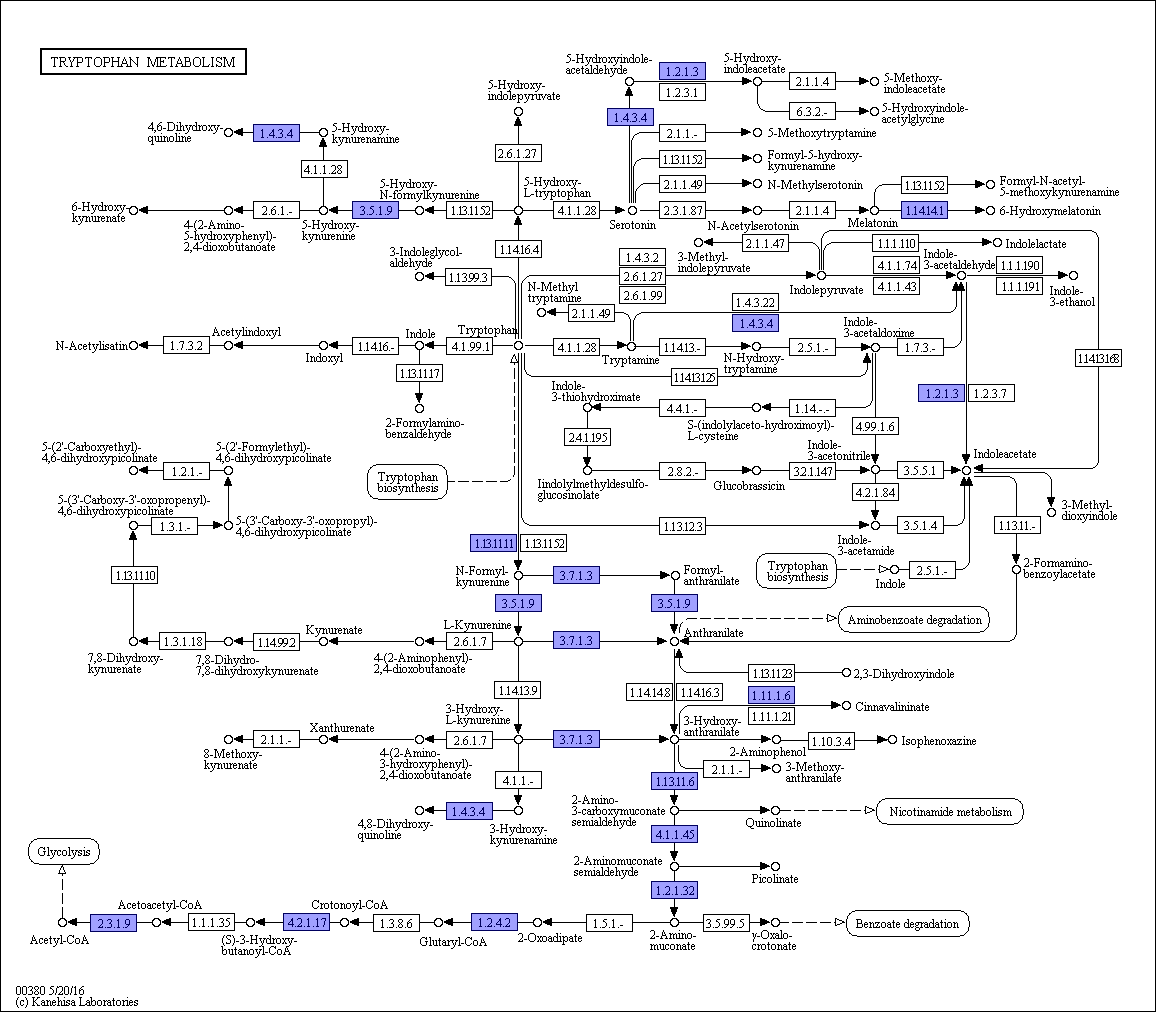


**Supplementary Figure 3.** Tryptophan Metabolism Pathway


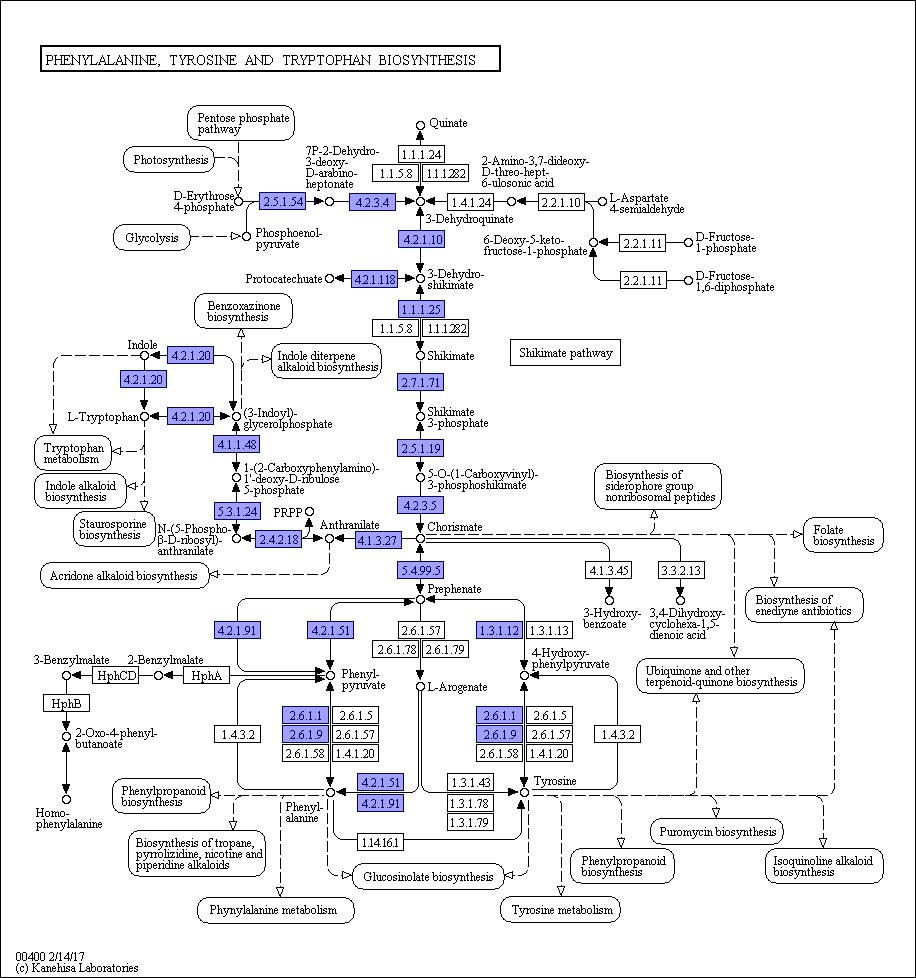


**Supplementary Figure 4.** Tryptophan Synthesis Pathway


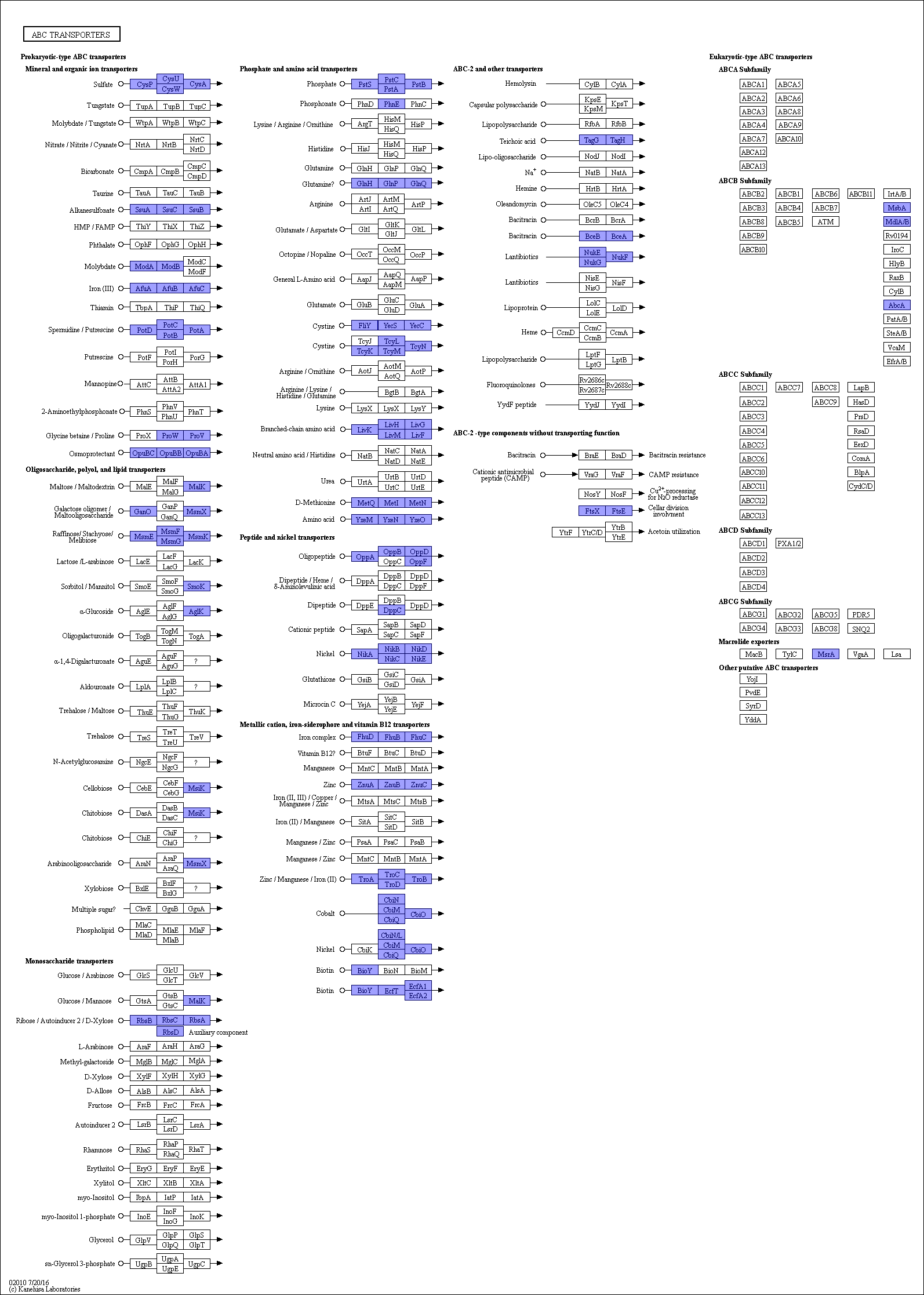


**Supplementary Figure 5.** Phosphate metabolism pathway


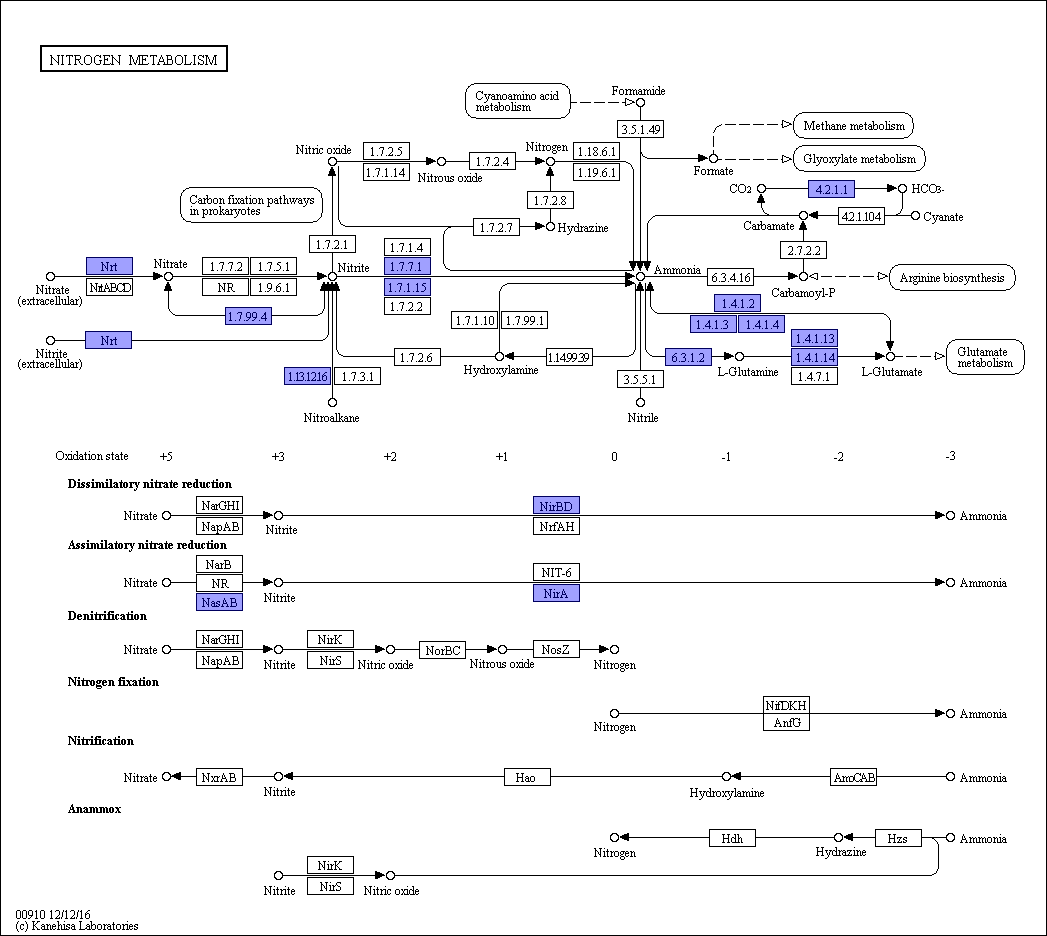


**Supplementary Figure 6.** Nitrogen metabolism pathway
